# *Staphylococcus succinus* 14BME20 Prevents Allergic Airway Inflammation by Induction of Regulatory T Cells via Interleukin-10

**DOI:** 10.3389/fimmu.2019.01269

**Published:** 2019-06-04

**Authors:** Jisun Song, Hui Xuan Lim, Arim Lee, Soojung Kim, Jong-Hoon Lee, Tae Sung Kim

**Affiliations:** ^1^Department of Life Sciences, College of Life Sciences and Biotechnology, Korea University, Seoul, South Korea; ^2^Department of Food Science and Biotechnology, Kyonggi University, Suwon, South Korea

**Keywords:** asthma, *Staphylococcus succinus*, regulatory T cells, dendritic cells, Interleukin-10

## Abstract

Asthma is a common chronic inflammatory disease, which is characterized by airway hyperresponsiveness (AHR), high serum levels of immunoglobulin (Ig)E, and recruitment of various inflammatory cells such as eosinophils and lymphocytes. Korean traditional fermented foods have been reported to exert beneficial effects against allergic diseases such as asthma and atopic dermatitis. In this study, we investigated whether *Staphylococcus succinus* strain 14BME20 (14BME20) isolated from doenjang, a traditional high-salt-fermented soybean food of Korea, exerts suppressive effects on allergic airway inflammation in a murine model. Mice were orally administered with 14BME20, then sensitized and challenged with ovalbumin as an allergen. Administration of the 14BME20 significantly suppressed AHR and influx of inflammatory cells into the lungs and reduced serum IgE levels. Moreover, the proportion of T helper type 2 (Th2) cells and the production of Th2 cytokines were decreased in 14BME20-treated mice, whereas dendritic cells (DCs) with tolerogenic characteristics were increased. In contrast, oral administration of 14BME20 increased the proportion of CD4^+^CD25^+^Foxp3^+^ regulatory T (Treg) cells and the level of interleukin (IL)-10 in 14BME20-treated mice. Furthermore, 14BME20 induced maturation of tolerogenic DCs, and 14BME20-treated DCs increased Treg cell population in a co-culture system of DCs and CD4^+^ T cells. The addition of a neutralizing anti-IL-10 mAb to the culture of cells that had been treated with 14BME20 decreased the enhanced Treg cell population, thereby indicating that 14BME20-treated DCs increase Treg cell population via DC-derived IL-10. These results demonstrate that oral administration of 14BME20 suppresses airway inflammation by enhancing Treg responses and suggest that the 14BME20 isolated from doenjang may be a therapeutic agent for allergic asthma.

## Introduction

Asthma is a T helper type 2 (Th2)-mediated inflammatory disease, characterized by high levels of immunoglobulin (Ig)E, airway hyperresponsiveness (AHR), mucus production by goblet cells, and recruitment of inflammatory cells, such as eosinophils and lymphocytes (1, 2). The pathogenesis of allergic asthma is related to innate immune cells, such as type 2 innate lymphoid cells and mast cells, and adaptive immune cells, such as Th2, Th17, and Th9 cells (3–6). On the other hand, regulatory T (Treg) cells induce airway tolerance by regulating immune responses induced by allergens (7–9).

In the past few decades, many studies have shown that probiotics have beneficial effects on the development of allergic asthma. For example, oral administration of *Lactobacillus rhamnosus* GG and *Bifidobacterium lactis* Bb-12 was shown to attenuate the development of airway inflammation by decreasing the recruitment of eosinophils and increasing the expression of transforming growth factor-β (TGF-β) and Foxp3 (10). Moreover, orally administered *Enterococcus faecalis* FK-23 inhibited allergic airway responses through suppression of Th17 cell development (11) and *Bifidobacterium infantis* CGMCC313-2 suppressed airway asthma by reducing the levels of interleukin (IL)-4 and IL-13 (12). These studies suggest that probiotics can alleviate allergic airway inflammation by regulating various immune functions.

Various probiotics isolated from Korean fermented foods have been reported to relieve allergic diseases through modulation of immune responses. For example, oral administration of heat-killed *Lactobacillus plantarum* KTCT3104 and *Lactobacillus curvatus* KTCT3767 isolated from kimchi was shown to inhibit allergic airway inflammation by reducing Th2 responses in the mediastinal lymph nodes (mLNs) and inducing Foxp3 expression in the intestines (13). In addition, *Weissella cibaria* WIKIM28 isolated from got kimchi suppressed the development of atopic dermatitis by inducing the generation of regulatory dendritic cells (DCs) and CD4^+^CD25^+^Foxp3^+^ Treg cells (14). Therefore, these studies suggest that probiotics isolated from Korean traditional fermented foods can alleviate allergic diseases by regulating immune functions. However, studies on the immune function of bacteria isolated from other fermented foods, except for kimchi, are insufficient. Doenjang is a traditional high-salt-fermented soybean food of Korea, and it is consumed with vegetables and meat. Recently, coagulase-negative staphylococci (CNS) have been isolated as a predominant bacterial group of doenjang (15). Jeong et al. (16) assessed the safety and technological properties of *Staphylococcus saprophyticus, Staphylococus succinus*, and *Staphylococcus xylosus* isolates, which were the predominant species among the CNS isolates. *S. succinus* strain 14BME20 (thereafter referred to as 14BME20) cleared the safety and functionality tests, and was selected as a potential starter culture candidate for soybean food fermentations. Furthermore, the complete genome sequence analysis of 14BME20 revealed that the strain does not encode any of the virulence factors found in the well-known pathogen *Staphylococcus aureus* (17).

In this study, we investigated whether 14BME20 isolated from doenjang regulates immune response and has beneficial effects on allergic diseases. We assessed allergic asthma features after oral administration of 14BME20 before allergen challenge. We found that oral administration of 14BME20 inhibited allergic airway inflammation features, including AHR, serum levels of IgE, and Th2 responses. In addition, IL-10 production and CD4^+^CD25^+^Foxp3^+^ Treg cell population increased in the peripheral lymph nodes of 14BME20-treated mice. Furthermore, treatment of DCs with 14BME20 induced tolerogenic DCs that produce IL-10, leading to an increase in CD4^+^CD25^+^Foxp3^+^ Treg cell population. These results indicate that 14BME20 protects against allergic asthma via induction of Treg responses.

## Materials and Methods

### Mice

Seven-weeks-old female BALB/c mice were purchased from Youngbio (Osan, Korea). All mice were maintained in specific-pathogen free facility in Korea University. All animal experiments were performed according to the Korea University Guidelines for the Care and Use of Laboratory Animals (approval No. KUIACUC-2015-48, 2016-174, 2017-109).

### Preparation of Bacteria

*Staphylococcus succinus* strain 14BME20 used in this study was isolated from doenjang, as previously described (15). 14BME20 was cultured in tryptic soy broth (TSB; Difco, Detroit, MI, USA) at 37°C for 16 h, and then the culture was diluted 1:100 in fresh TSB and cultured until optimal growth. The cultured bacteria were washed twice with sterile phosphate-buffered saline (PBS). For *in vivo* experiment, the washed bacteria inactivated by heating at 100°C for 30 min, then live 14BME20 and heat inactivated 14BME20 were immediately administered to the mice via the oral route. For *in vitro* experiment, heat-inactivated 14BME20 were stored at −20°C until use.

### Induction of Airway Inflammation and Oral Administration of *Staphylococcus succinus* 14BME20

Airway inflammation was induced as previously described (13). Briefly, 14BME20 was orally administered into the mice by oral zonde needle as much as 5 × 10^7^ CFU per mouse every other day from day 0 to day 20. The mice received 100 μg ovalbumin (OVA) (Grade V; Sigma-Aldrich, St. Louis, MO, USA) dissolved in 2 mg of aluminum hydroxide at 7 and 14 days by intraperitoneal injection. One week after the second OVA injection, mice were challenged with 3% (day 21 to 23) and 10% (day 24) OVA for 30 min in an exposure chamber using Pulmo-Aid Nebulizer (Devilbiss, Sunrise Medical Cor. USA). The mice were anesthetized with avertin (2.5% wt/vol in PBS) at 48 h after the last OVA challenge, BAL fluids, lung, mediastinal lymph nodes (mLNs), mesenchymal lymph nodes (MLNs), and spleen were collected.

### Assessment of Airway Hyperresponsiveness

Airway hyperresponsiveness was assessed after 24 h the last OVA challenge. The mice were exposed to various concentrations (6.25, 12.5, 25, 50, and 100 mg/ml) methacholine (Mch) (acetyl β-methylcholine chloride; Sigma-Aldrich). Afterward, pause of breathing (penh) value was measured for 3 min using the OCP-3000 (Allmedicus, Seoul, Korea), a non-invasive whole body plethysmography.

### Bronchoalveolar Lavage (BAL) Fluids Analysis and Lung Histology

BAL fluids were obtained by tracheal infusions of 1.5 ml PBS. Total BAL fluid cells were stained using trypan blue and viable cells were counted using a hemocytometer as previously described (18), and supernatants were stored at −80°C until cytokine analysis. The cell numbers of differential cells such as eosinophils and lymphocytes were counted after cytocentrifuge and Giemsa staining. For histological analysis, the lung tissues were fixed in 4% paraformaldehyde solution, and embedded in OCT compound (Tissue-Tek®, SAKURA, USA). OCT compound-embedded lung tissue were sectioned 5 μm and were stained with haematoxylin and eosin. Lung histologic feature was examined by light microscope (Olympus IX71; Olympus, Tokyo, Japan) and visualized by using RS Image software. The inflammatory score was assessed peribronchial and perivascular infiltration of inflammatory cells. The score was established as previously described (19, 20). 0, no detectable inflammation; 1, few cells (bronchus or vascular were surrounded by a few inflammatory cells); 2, a bronchus and vascular were surrounded by a layer of one cells; 3, a bronchus and vascular were surrounded by layer of two to four cells; 4, bronchus and vascular were surrounded by layer of more than four cells.

### Preparation of Mediastinal Lymph Nodes, Mesenteric Lymph Nodes, and Splenocytes

The mLNs, MLNs, and spleen were isolated from asthmatic mice. The mLNs and MLNs were minced with slide glass and then filtered using 40 μm cell strainer (BD Biosciences, San Diego, USA) and washed 2 times with RPMI1640 (Thermo Fisher Scientific, inc., Waltham, MA, USA) containing 10% heat-inactivated fetal bovine serum (FBS) (Capricon; Capricorn Scientific GmbH, Germany), 50 μM 2-mercaptoethanol (2-ME; Sigma-Aldrich; Merck Millipore, Darmstadt, Germany), 10 mM HEPES (Corning; Thermo Fisher Scientific, inc.), 100 U/ml penicillin, and 100 μg/ml streptomycin (both from Invitrogen; Thermo Fisher Scientific, Inc.). The spleens were also minced with slide glass and then filtered using a 70 μm cell strainer (BD Biosciences) and the red blood cells (RBC) in splenocytes were treated with RBC lysis buffer containing 0.15 M NH_4_Cl, 1 mM KHCO_3_ and 0.1 mM EDTA and washed 2 times with RPMI1640. Single cells were seeded at 3 × 10^6^ cells/ml in 24-well plate and re-stimulated with 100 μg/ml OVA and cultured for 4 days at 37°C with 5% CO_2_ incubator.

### Preparation of Single Cell Suspensions From Lungs

Single cell suspensions from mouse lungs were prepared, as described previously (21). Briefly, the lungs isolated from anesthetized asthmatic mice were placed in RPMI 1640, and gently minced on ice using a 70 μm strainer (BD Biosciences) and a 10 ml syringe piston. The cells were then incubated in RPMI 1640 containing 0.5 mg/ml collagenase D (Roche Diagnostics, Basel, Switzerland) and 20 μg/ml DNase I (Roche Diagnostics) at 37°C for 2 h with gentle stirring. The digested lungs were filtered through a 70 μm cell strainer, and then RBC were removed by using RBC lysis buffer. Single cells were seeded at 1 × 10^6^ cells/ml in 24-well plate with 100 μg/ml OVA and cultured for 4 days at 37°C with 5% CO_2_ incubator.

### Measurement of Immunoglobulins and Cytokines

Immunoglobulins and cytokines were measured via enzyme-linked immunosorbent assay (ELISA), as previously described (13, 22). Serum was obtained via cardiac puncture and stored at −20°C until analysis. For the detection of OVA-specific IgE, each well of the plate (Costar 3590, USA) was coated with 10 μg/ml of OVA and detected using biotinylated anti-mIgE EM95 provided by R. Coffman (DNAX Research Institute, Palo Alto, USA). For the detection of OVA-specific IgG1 and OVA-specific IgG2a, wells of plate (Costar 3590) were coated with 10 μg/ml of OVA and detected with anti-OVA IgG1 6C1, anti-OVA IgG2a 3A11, respectively. Specific mAbs for cytokine detection were used as following; for IL-4, 11B11 (anti-mIL-4 mAb) and biotinylated anti-mIL-4 BVD6-24G2 (both obtained from M. Howard, DNAX Research Institute); for IL-5, TRFK5 (anti-mIL-5 mAb) and biotinylated anti-mIL-5 TRFK4 (both from eBioscience, San Diego, USA) were used. For the detection of IL-13, IL-10, and IL-12p70 were measured using ELISA Ready-SET-Go!® (eBioscience) according to the manufacturer's instructions.

### Generation and Stimulation of Mouse Bone Marrow-Derived Dendritic Cells

Mouse bone marrow-derived dendritic cells (DCs) were generated, as described previously (23). In brief, the femurs and tibiae of 8-week-old female BALB/c mice were cut and flushed out with ice-cold RPMI 1640 using a syringe. Thereafter, cells were dissociated by pipetting and then filtered using a 70 μm cell strainer to remove debris. The RBCs were lysed using RBC lysis buffer. Lastly bone marrow cells (5 × 10^6^ cells/10 ml) were seeded in petri dishes with complete RPMI 1640 containing 10 ng/ml GM-CSF (ProSpec, Rehovort, Israel) and cultured at 37°C in a 5% CO_2_ incubator. The fresh RPMI1640 with GM-CSF was added on day 3 and day 5. At day 7, loosely adherent cells were harvested and seeded in 24-well plate at 2 × 10^6^ cells/well. Then, the cells were stimulated with heat-inactivated 14BME20 or lipopolysaccharide (LPS) (100 ng/ml; Sigma-Aldrich) for 24 h.

### Isolation and Characterization of Mucosal Dendritic Cells

Mice were orally administered with 200 μl PBS or bacterial suspension containing 5 × 10^7^ CFU of 14BME20 for 10 days. Mice were sacrificed and mucosal dendritic cells (MDCs) were isolated from lamina propria of small intestines, as described previously (24). In brief, the small intestines from the PBS- or 14BME20-treated mice (*n* = 3) were washed in cold PBS, and feces, fat tissues and Peyer's patches were removed. Afterwards, the intestines were cut into pieces (2–3 cm) and washed with cold PBS, and then incubated in RPMI 1640 containing 1 mM ethylenediaminetetraacetic acid (EDTA) with gentle stirring at 37°C for 15 min, followed by washing with warm PBS. Subsequently, the tissues were finely cut and incubated in RPMI 1640 containing 0.1 mg/ml of collagenase D (Roche Diagnostics) and 37°C for 30 min with gentle stirring. The cells were collected using 70 μm cell strainer, and the cells were centrifuged at 400 × g for 3 min at 4°C. Total lamina propria cells were isolated with 40 and 85% Percoll gradient media (GE Healthcare Life Science, Little Chalfont, UK) by gradient centrifugation. Total lamina propria cells were divided to two groups. One group was immediately stained with fluorescent antibodies for phenotypic analysis of DCs, and the other group was purified by CD11c magnetic bead (MACS; Miltenyi Biotec GmbH, Bergisch Gladbach, Germany) for mRNA analysis.

### Reverse Transcription-Polymerase Chain Reaction (RT-PCR) Analysis

BMDCs (2 × 10^6^ cells/well) were treated for 24 h with 14BME20 or LPS. Total RNA from the BMDCs was extracted with RiboEX total RNA kit (GeneAll Biotechnology, Seoul, Korea) and reverse transcribed into cDNA using a RocketScript™ Reverse Transcriptase kit (Bioneer Corporation, Daejeon, Korea). After cDNA was synthesized, the cDNA was amplified by PCR using AccuPower® PCR PreMix (Bioneer Corporation). The sequences of the RT-PCR primers used in this study were as follows: murine TGF-β forward, 5′-TAT AGC AAC AAT TCC TGG CG-3′ and reverse, 5′-TCC TAA AGT CAA TGT ACA GC-3′; murine IL-10 forward, 5′-AGA AAT CAA GGA GCA TTT GA-3′ and reverse, 5′-CTG CAG GTG TTT TAG CTT TT-3′; murine IDO forward, 5′-TTA TGC AGA CTG TGT CCT GGC AAA CTG-3′ and reverse, 5′-TTT CCA GCC AGA CAG ATA TAT GCG GAG-3′; murine COX-2 forward, 5′-GTG GAA AAA CCT CGT CCA GA-3′ and reverse, 5′-TGA TGG TGG CTG TTT TGG TA-3′; murine IL-12p40 forward, 5′-TTA TGC AAA TTG TGA GCT TG-3′ and reverse, 5′-CCT TTG CAT TGG ACT TCG GTA G-3′; murine β-actin forward, 5′-CGC AGA GTC TCG CCA TTA TG-3′ and reverse, 5′-TAA AAC GCA GCT CAG TAA CAG TCC G-3′. After cDNA amplification, the products were separated on 1.5% (w/v) agarose gels and stained with StainingSTAR (DyneBio, Gyeonggi-do, Korea). The relative expression of cytokines was analyzed by Image J software.

### *In vitro* Polarization of CD4^+^ T Cells With DCs

The DCs cultured for 7 days were pulsed with 100 μg/ml OVA for 2 h and then treated with 14BME20 (1:10 cell to bacteria ratio) or LPS (100 ng/ml) for 24 h. WT mice were injected by subcutaneous with OVA (100 μg/mouse) and 7 days later CD4^+^ T cells were obtained from the lymph nodes of OVA-injected mice by magnetic bead purification (MACS). Naïve CD4^+^ T cells were mixed with OVA-pulsed immature DCs or OVA-pulsed 14BME20- or OVA-pulsed LPS-treated DCs at 1:10 ratio. After 4 days, the cells were re-stimulated for 5 h with PMA (50 ng/ml, Sigma-Aldrich), ionomycin (1 μg/ml, Sigma-Aldrich) and brefeldin A (Golgiplug, 1 μg/ml; BD Biosciences). After 5 h, the cells were harvested and stained with fluorescent antibodies for flow cytometric analysis, and the supernatant was collected for determining cytokine levels. To determine the effect of IL-10 on the induction of Treg cells, neutralizing mAb against IL-10 (eBioscience) and TGF-β (R&D Systems; Minneapolis, MN, USA) was added to the DC-CD4^+^ T cell co-cultures.

### Flow Cytometric Analysis

For DCs phenotypic analysis, 14BME20- or LPS-treated cells were harvested, and were washed with FACS buffer (containing 0.5% fetal bovine serum and 0.05% sodium azide in PBS). Cells were block the Fc receptors using mIgG (Sigma-Aldrich) and then washed and stained with APC-conjugated anti-CD11c (BU15; eBioscience) and PE-conjugated anti-CD40 (3/23; BD Biosciences), anti-CD86 (GL1; BD Biosciences), anti-I-A^d^ (34-5-3S; BD Biosciences), and anti-CD274 (MIH5; BD Biosciences) and FITC-conjugated anti-CD103 (2E7, ebioscience) or isotype controls (eBioscience, BD Biosciences) for 15 min. For intracellular staining, CD4^+^ T cells stimulated with PMA, ionomycin, and brefeldin A were harvested and stained FITC-conjugated CD4 mAb (RM4-5; BD Biosciences), and APC-conjugated CD25mAb (PC61; BD Biosciences) for 15 min. Subsequently, the cells were fixed using Cytofix/Cytoperm kit (BD Biosciences) for 20 min. Afterward, the fixed cells were then stained with PE-conjugated anti-IL-4 (11B11; BD Biosciences), anti-IL-13 (eBio13A; eBioscience), anti-Foxp3 (FJK-16s; eBioscience) or APC-conjugated anti-IL-17 (eBio17B7; eBioscience), anti-IFN-γ (XMG1.2; BD Biosciences), Percp/cy5.5-conjugated anti-TGF-β1 (TW7-16B4, Biolegend) for 1 h. The stained cells were detected using FACS Calibur and FACS accuri (BD Biosciences) and the data were analyzed using Cell Quest and Accuri C6 software (BD Biosciences).

### Statistical Analysis

Data are expressed as the means ± standard error of the mean (SEM) and all experiments were conducted at least three times independently. Statistical analysis was performed using SigmaPlot version 12.5 (Systat Software Inc., Washington, USA), and differences between experimental and control groups were analyzed using Student's *t*-test or one-way analysis of variance (ANOVA). A *p* < 0.05 was considered to be statistically significant.

## Result

### Oral Administration of 14BME20 Prevents the Development of Airway Inflammation in a Murine Asthma Model

**Figure 1:**
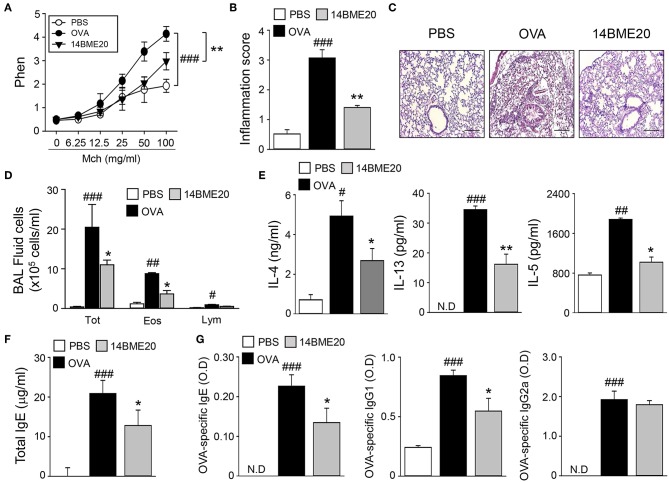
14BME20 suppresses lung airway inflammation. Mice were orally administered with PBS or 14BME20, and then sensitized and challenged with OVA. **(A)** The AHR was evaluated by methacholine administration after 24 h of the last OVA challenge. The graph indicates Penh value. **(B,C)** The lung sections were stained with hematoxylin and eosin. All tissue images were obtained at 100X magnification. The scale bar shows 500 μm. The inflammatory score was assessed by measuring the infiltration of inflammatory cells into the peribronchial and perivascular tissues. **(D)** Total inflammatory cells, eosinophils and lymphocytes in the BAL fluid were counted using trypan blue stain or Giemsa stain, respectively. **(E)** The level of Th2 cytokines in the BAL fluid was measured by ELISA. **(F,G)** The level of immunoglobulins in serum was measured by ELISA. Data are representative of four independent experiments, and bar graphs represent means ± SEM. ###*p* < 0.001, ##*p* < 0.01, #*p* < 0.05 vs. the PBS group, **p < 0.01, *p < 0.05 vs. the OVA group. N.D., not detected.

To examine whether 14BME20 isolated from doenjang can be useful in preventing the development of allergic airway inflammation, we utilized a well-established model of experimental asthma. Mice were orally administered with 14BME20 every other day and were sensitized to OVA/alum via intraperitoneal injection, and then challenged with PBS or OVA via aerosol for 4 days. As shown Figure 1A, 14BME20-treated mice exhibited significant suppression of the AHR compared with OVA control mice. We also found that recruitment of inflammatory cells into the peribronchial and perivascular tissues was markedly suppressed in mice treated with 14BME20 compared with that in OVA control mice (Figures 1B,C). In addition, as compared to OVA control mice, mice orally administered with 14BME20 showed a dramatically reduced number of total cells, eosinophils, and lymphocytes, and also exhibited decreased production of Th2-related cytokines, such as IL-4, IL-13, and IL-5 in BALFs (Figures 1D,E). Moreover, the serum level of total IgE, one of the hallmarks of asthma, was significantly decreased in 14BME20-treated mice compared to that in OVA control mice (Figure 1F). Furthermore, the levels of OVA-specific IgE and IgG1 associated with Th2 responses were markedly reduced in 14BME20-treated mice, whereas the level of IgG2a associated with Th1 responses was not affected by 14BME20 treatment (Figure 1G). Additionally, heat-killed 14BME20 also dramatically inhibited airway inflammation as did live 14BME20 (Supplementary Figure 1). Collectively, these results indicate that oral administration of 14BME20 suppresses the development of allergic airway inflammation in mice.

### 14BME20 Inhibits Th2 Responses During Allergic Airway Inflammation

**Figure 2:**
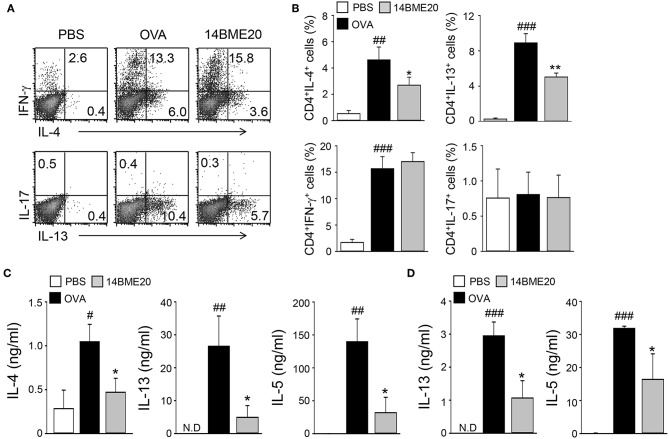
14BME20 reduces Th2 immune responses during allergic airway inflammation. Mice were orally administered with PBS or 14BME20, and then challenged with OVA. Following this, CD4^+^ T cells were isolated from the mediastinal LNs and lungs, then re-stimulated with OVA (100 μg/ml) for 4 days. **(A,B)** The proportion of CD4^+^ T cell subsets was analyzed by flow cytometry. **(C,D)** Cytokine production in the mediastinal LNs and lungs was measured by ELISA. Data are representative of more than three independent experiments, and bar graphs represent means ± SEM. ###*p* < 0.001, ##*p* < 0.01, #*p* < 0.05 vs. the PBS group. **p < 0.01, *p < 0.05 vs. the OVA group. N.D., not detected.

As T cells are known to play a role in the pathogenesis of airway inflammation (25), we investigated whether administration of 14BME20 affects CD4^+^ T cell-mediated immune response during airway inflammation. Mice were orally administered with PBS or 14BME20 and challenged with OVA. Then, the mLNs were isolated from asthmatic mice and re-stimulated with OVA. As shown Figures 2A,B, mice treated with 14BME20 showed remarkably decreased levels of IL-4- and IL-13-expressing CD4^+^ T cell populations compared to OVA control mice. However, oral administration of 14BME20 did not affect the populations of interferon (IFN)-γ- and IL-17-expressing CD4^+^ T cells. Next, we measured the cytokine levels produced by the mLNs and the lungs to confirm that 14BME20 administration reduced Th2 responses. The levels of Th2 cytokines, such as IL-4, IL-13, and IL-5 were markedly lower in mice administered 14BME20 than those in OVA control mice (Figures 2C,D). Under naïve condition, 14BME20 didn't affect the percentage of Th2 cell population in the lungs. However, when the animal is exposed to the allergen, Th2 cell population was decreased in the lungs of 14BM20-treated mice (Supplementary Figure 2). Consistent with these results, the levels of IL-4- and IL-13-expressing CD4^+^ T cell populations were dramatically reduced in the splenocytes of 14BME20-treated mice compared to those in the splenocytes of OVA control mice. Moreover, the levels of Th2 cytokines, IL-4, IL-13, and IL-5, were dramatically decreased in the splenocytes of mice orally administered with 14BME20 compared to that in the splenocytes of OVA control mice. However, oral administration of 14BME20 did not affect the levels of both IFN-γ- and IL-17-expressing CD4^+^ T cell populations in the splenocytes, as in mLNs (Supplementary Figure 3). Taken together, these data suggest that 14BME20 inhibited allergic airway inflammation by suppressing Th2 responses.

### Oral Administration of 14BME20 Increases Treg Responses in Asthmatic Mice

**Figure 3:**
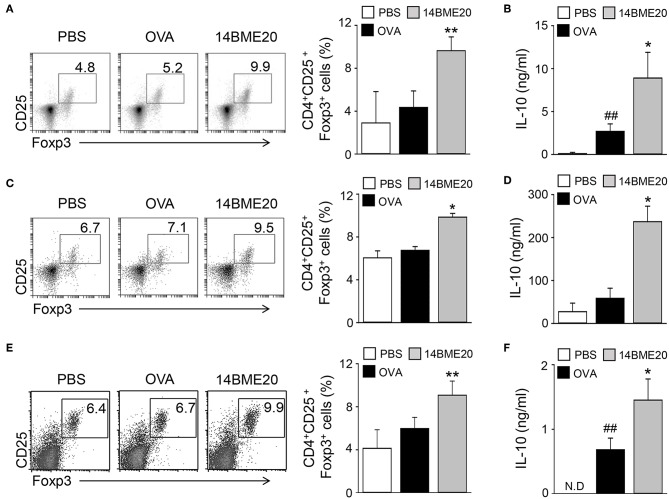
14BME20 increases the proportion of Treg cell population and the level of IL-10 in asthmatic mice. The mediastinal LNs **(A,B)**, lungs **(C,D)**, and MLNs **(E,F)** were obtained from mice exposed to OVA, followed by administration with 14BME20 or PBS, and then re-stimulated with OVA (100 μg/ml) for 4 days. **(A,C,E)** The proportion of Treg cells was determined by flow cytometry. **(B,D,F)** The level of IL-10 was measured by ELISA. Data are representative of three independent experiments, and bar graphs represent means ± SEM. ##*p* < 0.01 vs. the PBS group. **p < 0.01, *p < 0.05 vs. the OVA group. N.D., not detected.

Many studies have proven that Treg cells induce airway tolerance by reducing Th1, Th2, and Th17 responses as well as T cell proliferation in allergic asthma (7–9, 26, 27). For example, Kearley et al. demonstrated that the adoptive transfer of OVA-specific CD4^+^CD25^+^ Treg cells to OVA-sensitized mice decreased the number of Th2 cells and the level of IL-5 and IL-13, whereas the level of IL-10 was increased (28). These results indicated that there is a correlation between Th2 and Treg cells. Additionally, previous studies have reported that oral administration of probiotics induces CD4^+^CD25^+^Foxp3^+^ Treg cells and increases the levels of IL-10 and TGF-β (10, 13, 14). Therefore, we investigated whether 14BME20, which inhibited Th2 responses, affects Treg responses in peripheral LNs, lungs, and MLNs of asthmatic mice. We isolated mLNs, lungs, and MLNs from asthmatic mice fed PBS or 14BME20 and re-stimulated with OVA for 4 days. The proportion of CD4^+^CD25^+^Foxp3^+^ Treg cells and the production of IL-10 were higher in the mLNs, lungs, and MLNs of mice treated with 14BME20 than those in OVA control mice (Figure 3). Under naïve condition, 14BME20 didn't affect CD4^+^CD25^+^Foxp3^+^ Treg cell population in the lungs. However, when the animal is exposed to the allergen, 14BME20 increased the percentages of CD4^+^CD25^+^Foxp3^+^ Treg cell population in the lungs (Supplementary Figure 2). In addition, the splenocytes of mice treated with 14BME20 showed an increased (not significant) proportion of CD4^+^CD25^+^Foxp3^+^ Treg cells and significantly increased production of IL-10 (Supplementary Figure 3). These results suggest that oral administration of 14BME20 induced differentiation of Treg cells and production of IL-10, leading to suppression of allergic responses.

### 14BME20 Treatment Induces tolerogenic DCs in the Asthmatic Mice

**Figure 4:**
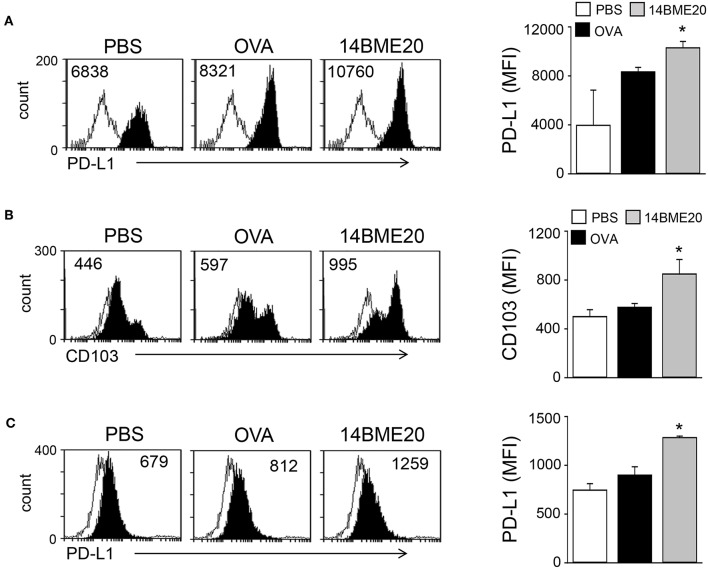
14BME20 induces tolerogenic DCs in the asthmatic mice. Lungs **(A)** and MLNs **(B,C)** were isolated from mice challenged with OVA following administration of PBS or 14BME20. Afterwards, the expression of PD-L1 and CD103 were analyzed by flow cytometry. Data are representative of two independent experiments, and bar graphs represent means ± SEM. *p < 0.05 vs. the OVA group.

Probiotics is known to induce differentiation of naïve CD4^+^ T cells into effector T cells via antigen-presenting cells, like DCs (29, 30). Especially, probiotics have previously demonstrated to produce Treg cells through tolerogenic DCs (14, 31). Therefore, we investigated whether 14BME20 induced tolerogenic DCs in the lungs and MLNs. After isolation of CD11c^+^ cells from lungs and MLN of asthmatic mice fed with 14BME20 or PBS alone, the expression of PD-L1 and CD103, which is known to induce and maintain Treg cells (32, 33), was examined on CD11c^+^ cells. As shown in Figure 4A, PD-L1 expression on the lung CD11c^+^ cells of the 14BME20-treated mice was significantly increased, compared to the OVA control mice. On the other hand, CD103^+^ DCs are known to require for the induction of Foxp3^+^ Treg cells in the intestines, and CD103^+^PD-L1^+^ DCs induce Treg cells in the MLN (34). Therefore, we also checked the expression of CD103 in the MLN cells. The expression of CD103 and PD-L1 was significantly increased in the MLN cells of 14BME20-treated mice, compared to the OVA control mice (Figure 4B).

**Figure 5:**
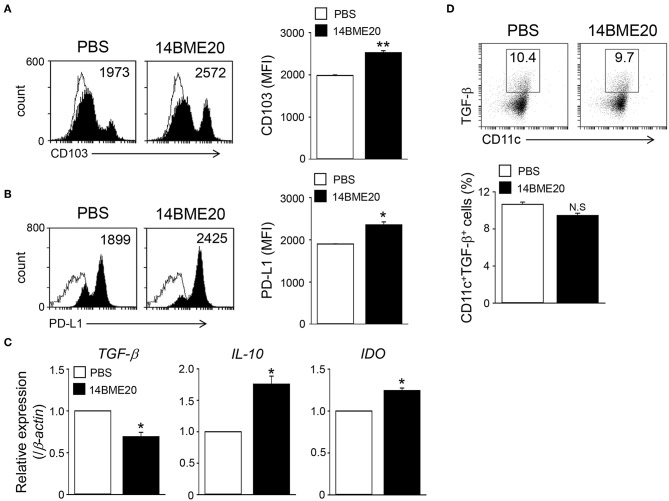
Mucosal DCs isolated from 14BME20-treated mice exhibit the enhanced tolerogenic phenotype. Mice were orally administered with 14BME20 or PBS for 10 days. At day 11, mice were sacrificed and mucosal DCs were isolated lamina propria of small intestines. **(A,B)** The expression of cell surface markers was analyzed by flow cytometry. **(C)** The levels of IL-10, TGF-β, and IDO in mucosal DCs were measured by qRT-PCR. **(D)** The percentage of CD11c^+^CD103^+^TGF-β^+^ cell population was analyzed by flow cytometry. Data are representative of two independent experiments (*n* = 3), and bar graphs represent means ± SEM. **p < 0.01, *p < 0.05 vs. the PBS-treated mice. N.S., not significant.

The intestinal CD103^+^ DCs play an important role in maintaining intestinal immune homeostasis (33, 35), and induce Treg cells (36). To investigate whether oral administration of 14BME20 also induces tolerogenic DCs in the intestines, mice were orally administered with 14BME20 or PBS for 10 days, and mucosal DCs were isolated from the lamina propria of 14BME20- or PBS-treated mice. As shown Figure 5A, the expression of CD103 on mucosal DCs of 14BME20-treated mice were significantly increased, compared to PBS-treated mice. Moreover, the PD-L1 expression, which is involved in inducing Treg cells in the MLN (34, 37), was markedly increased in mucosal DCs of 14BME20-treated mice (Figure 5B). The expression of IL-10 and IDO was also increased in mucosal DCs of 14BME20-treated mice, compared to PBS-treated mice. In contrast, TGF-β expression was reduced by 14BME20 treatment (Figures 5C,D). These results indicate that oral administration of 14BME20 induced tolerogenic DCs during allergic airway inflammation and promoted Treg cell differentiation.

### 14BME20 Generates Tolerogenic DCs *in vitro*

**Figure 6:**
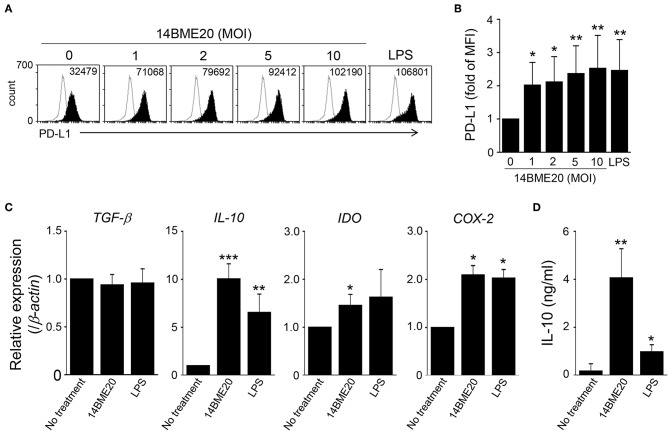
14BME20 generates tolerogenic DCs. BMDCs were treated with various MOIs (0, 1, 2, 5, and 10) of 14BME20 or LPS (100 ng/ml) for 24 h. **(A,B)** The expression of PD-L1 was detected by flow cytometry. **(C)** The expression of immunoregulatory molecules was analyzed by qRT-PCR. **(D)** The level of IL-10 was measured by ELISA. Data are representative of three independent experiments, and bar graphs represent means ± SEM. ***p < 0.001, **p < 0.01, *p < 0.05 vs. the negative control group (no treatment).

Since tolerogenic DCs in the asthma model were induced by oral administration of 14BME20, we further investigated the characteristics of tolerogenic DCs induced by 14BME20 by using BMDCs. The BMDCs were treated with 14BME20 or LPS for 24 h, and the markers of tolerogenic DCs were identified. The expression of PD-L1 was remarkably enhanced in 14BME20-treated DCs compared with that in control DCs (Figures 6A,B). In addition, 14BME20 treatment significantly increased the mRNA expression of IL-10, IDO, and COX-2, which are known to be expressed in tolerogenic DCs (Figure 6C), and also markedly increased IL-10 production (Figure 6D). However, the expression of TGF-β remained unchanged in DCs treated with 14BME20. These results suggest that 14BME20 generated tolerogenic DCs. Additionally, 14BME20 also affected the activation of DCs. The levels of the activation markers, CD40, CD86, and MHC-II, as well as the level of IL-12p70 were significantly up-regulated in 14BME20-treated DCs compared to those in control DCs (Supplementary Figure 4). Collectively, these results indicate that 14BME20 generated tolerogenic DCs and also induced DC activation.

### 14BME20 Induces Treg Cell Differentiation Through IL-10

**Figure 7:**
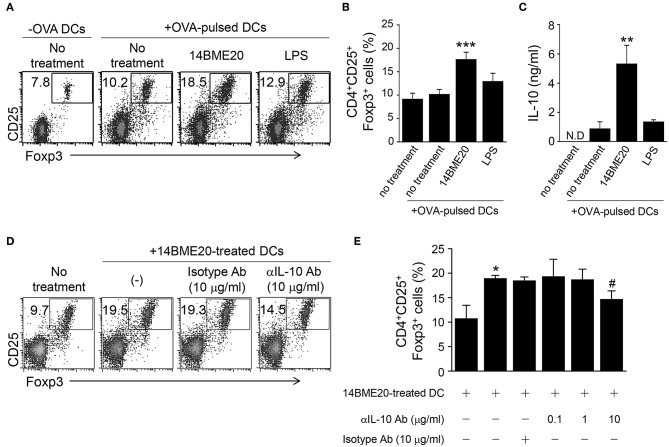
14BME20-treated DCs increase Treg cell population through IL-10. DCs were treated for 2 h with OVA (100 μg/ml). The OVA-pulsed DCs were treated with 14BME20 (MOI 10) or LPS for 24 h and co-cultured with naïve CD4^+^ T cells for 4 days. **(A,B)** The proportion of Treg cells was analyzed by flow cytometry. **(C)** The level of IL-10 in the co-culture supernatant was measured by ELISA. **(D,E)** The OVA-pulsed DCs treated with 14BME20 were incubated for 30 min with IL-10-neutralizing Ab, and then co-cultured for 4 days with naive CD4^+^ T cells. The proportion of Treg cells was analyzed by flow cytometry. Data are representative of three independent experiments, and bar graphs represent means ± SEM. ***p < 0.001, **p < 0.01, *p < 0.05 vs. the negative control (no treatment). #*p* < 0.05 vs. the 14BME20-treated group. N.D., not detected.

Because tolerogenic DCs are known to induce differentiation of Treg cells (38), we investigated whether 14BME20-treated DCs also promoted Treg cell differentiation. First, DCs were pulsed with OVA for 2 h and OVA-pulsed DCs were treated with 14BM20 or LPS for 24 h. Afterwards, 14BME20- or LPS-treated OVA-pulsed DCs were co-cultured with naive CD4^+^ T cells for 4 days. As shown in Figures 7A,B, 14BME20-treated DCs showed a markedly increased proportion of CD4^+^CD25^+^Foxp3^+^ Treg cells compared with control DCs. Moreover, 14BME20-treated DCs showed increased production of IL-10 compared with control DCs (Figure 7C). However, other CD4^+^ T cell subsets, including IFN-γ-, IL-4-, and IL-17-expressing CD4^+^ T cells, were not affected by 14BME20 treatment (Supplementary Figure 5). These data show that 14BME20 induced the differentiation of Treg cells and enhanced the production of IL-10. Next, we investigated whether the increase of Treg cell population by 14BME20 is IL-10 dependent. For this purpose, the 14BME20-treated OVA-pulsed DCs were treated with a neutralizing anti-IL-10 mAb, and then co-cultured with naive CD4^+^ T cells for 4 days. Addition of anti-IL-10 mAb to the co-culture of 14BME20-treated DCs and CD4^+^ T cells dramatically suppressed the differentiation of Treg cells (Figures 7D,E). Although 14BME20 had no effect on TGF-β expression, TGF-β in the presence of IL-10 may affect Treg cell differentiation when CD4^+^ T cells were co-cultured with 14BME20-treated DCs. Therefore, naïve CD4^+^ T cells were co-cultured with 14BME20-treated OVA-pulsed DCs in the absence or presence of neutralizing anti-IL-10 and/or anti-TGF-β mAbs. The percentage of Treg cell population in the co-culture treated with both anti-IL-10 and anti-TGF-β mAbs was similar to that treated with anti-IL-10 mAb alone (Supplementary Figure 6), indicating that 14BME20-induced Treg cell differentiation is IL-10-dependent.

## Discussion

In this study, we demonstrated that 14BME20 isolated from doenjang protected allergic asthma. Oral administration of 14BME20 significantly attenuated AHR, production of Th2 cytokines in BAL fluids, and accumulation of inflammatory cells in the lungs. In addition, the proportion of Th2 cells and the level of Th2 cytokines were decreased in the peripheral LNs of mice treated with 14BME20. In contrast, the level of Treg cell population and production of IL-10 were enhanced in the peripheral LNs and MLNs of mice administered with 14BME20. This study is the first to demonstrate the potential of CNS inhibiting allergic airway inflammation by modulating immune response to allergen.

Many studies have reported that probiotics have beneficial effects in regulating allergen-mediated immune responses in allergic diseases. Oral administration of *L. plantarum* KTCT3104, *L. curvatus* KTCT3767, and *Lactobacillus reuteri* ATCC23272 was shown to alleviate allergic asthma by inhibiting AHR and reducing the production of Th2 cytokines, such as IL-4 and IL-5 in BAL fluids (13, 39). Another study demonstrated that mice administered with *L. rhamnosus* GG and *L. rhamnosus* (Lcr35) showed decreased levels of Th2 cytokines, such as IL-4 and IL-5, as well as immunoglobulins associated with Th2 responses via CD4^+^CD25^+^Foxp3^+^ Treg cells (10, 40). Similar to this, our study showed that oral administration of 14BME20 significantly suppressed Th2 responses, such as the populations of CD4^+^IL-4^+^ and CD4^+^IL-13^+^ cells and production of Th2 cytokines, whereas the population of CD4^+^CD25^+^Foxp3^+^ Treg cells and IL-10 production were increased in the peripheral LNs and the lungs (Figures 2, 3). Therefore, these results suggest that 14BME20 ameliorated allergic airway inflammation by reducing Th2 responses and inducing Treg responses.

Oral administration of probiotics affects intestinal immunity by inducing Treg cells or immunosuppressive cytokines, such as IL-10 and TGF-β. For instance, oral administration of *Clostridium butyricum* CGMCC0313-1 has been shown to increase the proportion of CD4^+^CD25^+^Foxp3^+^ Treg cells in MLNs (41). Additionally, mice orally administered with *Weissella cibaria* WIKIM28 isolated from got kimchi showed increased proportions of CD4^+^CD25^+^Foxp3^+^ Treg cells and IL-10 production in the MLNs (14). In another study, oral treatment with *Lactobacillus brevis* SBC8803 markedly increased IL-10 and TGF-β production in the payer's patch (42). Similarly, our data showed that CD4^+^CD25^+^Foxp3^+^ Treg cell population and IL-10 production in the MLN of 14BME20-treated mice were increased (Figure 3). Thus, our data clearly demonstrate that oral administration of 14BME20 can induce a Treg response and has beneficial effects on intestinal immunity. Furthermore, CD4^+^CD25^+^Foxp3^+^ Treg cell population was also increased in the lungs of 14BME20-treated mice (Figure 3), indicating that Treg cells increased in the intestines exposed to 14BME20 can migrate to other organs and regulate the immune responses.

Although most probiotics have been reported to mitigate allergic disease through Treg cells, the mechanism by which they induce Treg cells is not yet clear. However, some probiotics have demonstrated the underlying mechanism by confirming that Treg cell is generated through tolerogenic DCs. Tolerogenic DCs are known to express PD-L1 on their surface. They primarily produce IL-10, TGF-β, IDO, and COX-2 and promote the differentiation of naïve CD4^+^ T cells into CD4^+^CD25^+^Foxp3^+^Treg cells (38, 43–45). Lim et al. demonstrated that *W. cibaria* WIKIM28 isolated from got kimchi induced tolerogenic DCs, which were identified by assessing the production of IL-10 and the expression levels of PD-L1 and ICOS-L on the cell surface. Later, it was demonstrated that *W. cibaria* WIKIM28-treated DCs promoted Treg cell differentiation in a co-culture system (14). In addition, other studies also proved that oral application of IRT5, which is a mixture of 5 probiotics, also generated tolerogenic DCs in the MLNs, draining LNs, and spleens. The population of CD4^+^Foxp3^+^ Treg cells was shown to be increased in CD4^+^ T cells co-cultured with IRT5-treated DCs (31, 46). Consistent with these studies, our data showed that the expression of PD-L1, IL-10, IDO, and COX-2, which are markers of tolerogenic DCs, was significantly up-regulated in 14BME20-treated DCs (Figure 6). As in the *in vitro* system, tolerogenic DCs were also induced by 14BME20 in both *in vivo* and *ex vivo* systems. The population of CD103^+^PDL1^+^ DCs and CD11c^+^ MHCII^hi^PDL1^+^ tolerogenic DCs were increased in the MLN and lungs of 14BME20-treated asthmatic mice, respectively (Figure 4). In the mucosal DCs isolated from the lamina propria of 14BME20-treated mice, the expression of CD103 and PDL1, which is known to be involved in promoting Treg cell differentiation, was increased, and the mRNA expression of IL-10 and IDO was also increased. However, in mucosal DCs, 14BME20 reduced TGF-β expression slightly or had no effect, as in the *in vitro* system (Figure 5). CD103^+^ DC is known to induce Treg cells by TGF-β- and retinoic acid-dependent ways (36, 47). 14BME20 used in our study did not affect the expression of TGF-β, a key factor that induces Treg cells in BMDC and mucosal DCs. However, Matteoli et al. (48) reported that IDOs expressed by CD11c^+^CD103^+^ DCs in lamina propria play a major role in Treg cell induction. In our study, 14BME20 increased the expression of IDO and IL-10 in both BMDC and mucosal DC (Figures 5, 6). Therefore, we speculate that 14BME20-induced increase of Treg cell population is dependent on IL-10 and IDO, not on TGF-β.

In the co-culture system, the proportion of CD4^+^IFN-γ^+^ cells remained unchanged in 14BME20-treated DCs (Supplementary Figure 5), but the proportion of CD4^+^CD25^+^Foxp3^+^ Treg cells as well as the levels of IL-10 were significantly enhanced (Figure 7). Thus, our data indicate that 14BME20 induced tolerogenic DCs, thereby promoting the differentiation of CD4^+^CD25^+^Foxp3^+^ Treg cells.

We also found that the generation of Treg cells by 14BME20 is dependent on IL-10. Treg cells were induced by immunosuppressive cytokines, such as, IL-10 and TGF-β. Our data showed that 14BME20 treatment increased the production of IL-10 about 30-fold, while it had no effect on TGF-β levels. The differentiation of Treg cells was suppressed by treatment with a neutralizing anti-IL-10 mAb. Thus, our data indicate that the differentiation of Treg cells induced by 14BME20 is mediated by IL-10.

Th1 cells are reported to maintain a balance between Th1 and Th2 and prevent Th2-mediated allergic disease. However, Th1 cells are also known to have no direct effects to inhibit Th2 cells. For example, excessive administration of antigen-specific Th1 cells does not inhibit airway hyperreactivity caused by Th2 cells (49). Since then, a number of studies have been conducted to demonstrate that Treg cells producing IL-10 have the ability to inhibit Th2 responses, such as IgE switching, eosinophilia, and AHR, to the antigen. For instance, Th1-like Treg cells have a regulatory effect dependent on IL-10. (50). Immunity to the antigen is dependent on IL-10 produced in DC, and this induces generation of CD4^+^ Treg cells that produce IL-10. (51). In addition, heat-killed *Mycobacterium vaccae* significantly reduces AHR and eosinophila via IL-10 and TGF-β (52). Consistent with these studies, we demonstrate that 14BME20 can inhibit eosinophilic airway inflammation by inducing tolerogenic DCs and Foxp3^+^ Treg cells that produce IL-10 and IDO.

Cell wall components or metabolites induce a variety of immune responses, including induction of Treg cells. Recently, cell surface β-glucan/galactan polysaccharides (CSGG) of *Bifidobacterium bifidum* have been identified as a major component inducing Treg cells (53). This CSGG induced IL-10-secreting Foxp3^+^ Treg cells through regulatory DCs that produce TGF-β and IL-10. In another study, polysaccharide A from *Bacteroides fragilis* is a key molecule that induces functional Treg cells that produce IL-10 during intestinal inflammation via TLR2 (54). In addition, γ-PGA, a bio-active metabolite of *Bacillus subtilis*, was also found to regulate the immune response. In particular, γ-PGA isolated from *Bacillus subtilis* of chungkookjang, a traditional Korean fermented food, is reported to alleviate atopic dermatitis by reducing the production of Th2 and Th17 cytokines (55). In our study, in addition to the treatment with live 14BME20, heat-inactivated 14BME20 similarly inhibited allergic airway inflammation by reducing the Th2 response. Therefore, it is expected that there will be a heat-stable component inducing tolerogenic DCs and Treg cells among cellular fractionates or metabolites, including cell membrane and cell surface of 14BME20.

In conclusion, our results show that oral administration of 14BME20 protects against allergic airway inflammation through IL-10-mediated Treg response. Therefore, 14BME20 can be used as a novel agent to prevent the development of allergic asthma. This study supports the potential of CNS species including *S. succinus* in the treatment of allergic diseases.

## Ethics Statement

All animal experiments were ethically performed according to the guidelines of the Korea University Institutional Animal Care and Use Committee (Seoul, Korea; approval no. KUIACUC-2015-48, 2016-174, 2017-109).

## Author Contributions

JS designed the experiment and performed all experiments for data collection and analyzed the data and drafted the manuscript. HL, AL, and SK performed *in vivo* experiments for data collection and also analyzed the data. TK conceived the study and participated in the design of the study. J-HL and TK analyzed the data and also wrote the manuscript. TK has full access to all the data in this study through financial support.

### Conflict of Interest Statement

The authors declare that the research was conducted in the absence of any commercial or financial relationships that could be construed as a potential conflict of interest.

## References

[1] Cookson W. The alliance of genes and environment in asthma and allergy. Nature. (1999) 402:B5–11. 10.1038/35037002 10586889

[2] Eder W Ege MJ von Mutius E. The asthma epidemic. N Engl J Med. (2006) 355:2226–35. 10.1056/NEJMra054308 17124020

[3] Scanlon ST McKenzie AN. Type 2 innate lymphoid cells: new players in asthma and allergy. Curr Opin Immunol. (2012) 24:707–12. 10.1016/j.coi.2012.08.009 22985480

[4] Bradding P Walls AF Holgate ST. The role of the mast cell in the pathophysiology of asthma. J Allergy Clin Immunol. (2006) 117:1277–84. 10.1016/j.jaci.2006.02.039 16750987

[5] Kudo M Ishigatsubo Y Aoki I. Pathology of asthma. Front Microbiol. (2013) 4:263. 10.3389/fmicb.2013.00263 24032029 PMC3768124

[6] Cosmi L Liotta F Maggi E Romagnani S Annunziato F. Th17 cells: new players in asthma pathogenesis. Allergy. (2011) 66:989–98. 10.1111/j.1398-9995.2011.02576.x 21375540

[7] Larché M. Regulatory T cells in allergy and asthma. Chest. (2007) 132:1007–14. 10.1378/chest.06-2434 17873195

[8] Lloyd CM Hawrylowicz CM. Regulatory T cells in asthma. Immunity. (2009) 31:438–49. 10.1016/j.immuni.2009.08.007 19766086 PMC3385348

[9] Curotto de Lafaille MA Lafaille JJ Graça L Mechanisms of tolerance and allergic sensitization in the airways and the lungs. Curr Opin Immunol. (2010) 22:616–22. 10.1016/j.coi.2010.08.014 20884192 PMC3900231

[10] Feleszko W Jaworska J Rha RD Steinhausen S Avagyan A Jaudszus A. Probiotic-induced suppression of allergic sensitization and airway inflammation is associated with an increase of T regulatory-dependent mechanisms in a murine model of asthma. Clin Exp Allergy. (2007) 37:498–505. 10.1111/j.1365-2222.2006.02629.x 17430345

[11] Zhang B An J Shimada T Liu S Maeyama K. Oral administration of *Enterococcus faecalis* FK-23 suppresses Th17 cell development and attenuates allergic airway responses in mice. Int J Mol Med. (2012) 30:248–54. 10.3892/ijmm.2012.1010 22641478

[12] Liu MY Yang ZY Dai WK Huang JQ Li YH Zhang J. Protective effect of *Bifidobacterium infantis* CGMCC313-2 on ovalbumin-induced airway asthma and β-lactoglobulin-induced intestinal food allergy mouse models. World J Gastroenterol. (2017) 23:2149–58. 10.3748/wjg.v23.i12.2149 28405142 PMC5374126

[13] Hong HJ Kim E Cho D Kim TS. Differential suppression of heat-killed lactobacilli isolated from kimchi, a Korean traditional food, on airway hyper-responsiveness in mice. J Clin Immunol. (2010) 30:449–58. 10.1007/s10875-010-9375-8 20204477

[14] Lim SK Kwon MS Lee J Oh YJ Jang JY Lee JH. *Weissella cibaria* WIKIM28 ameliorates atopic dermatitis-like skin lesions by inducing tolerogenic dendritic cells and regulatory T cells in BALB/c mice. Sci Rep. (2017) 7:40040. 10.1038/srep40040 28067304 PMC5220369

[15] Jeong DW Kim HR Jung G Han S Kim CT Lee JH. Bacterial community migration in the ripening of doenjang, a traditional Korean fermented soybean food. J Microbiol Biotechnol. (2014) 24:648–60. 10.4014/jmb.1401.01009 24548930

[16] Jeong DW Lee B Her JY Lee KG Lee JH. Safety and technological characterization of coagulase-negative staphylococci isolates from traditional Korean fermented soybean foods for starter development. Int J Food Microbiol. (2016) 236:9–16. 10.1016/j.ijfoodmicro.2016.07.011 27427871

[17] Jeong DW Lee JH. Complete genome sequence of *Staphylococcus succinus* 14BME20 isolated from a traditional Korean fermented soybean food. Genome Announc. (2017) 5:e01731–16. 10.1128/genomeA.01731-16 28254985 PMC5334592

[18] Arnold IC Dehzad N Reuter S Martin H Becher B Taube C. *Helicobacter pylori* infection prevents allergic asthma in mouse models through the induction of regulatory T cells. J Clin Invest. (2011) 121:3088–93. 10.1172/JCI45041 21737881 PMC3148731

[19] Tong J Bandulwala HS Clay BS Anders RA Shilling RA Balachandran DD. Fas-positive T cells regulate the resolution of airway inflammation in a murine model of asthma. J Exp Med. (2006) 203:1173–84. 10.1084/jem.20051680 16618792 PMC2121201

[20] Tamaru S Mishina H Watanabe Y Watanabe K Fujioka D Takahashi S. Deficiency of phospholipase A2 receptor exacerbates ovalbumin-induced lung inflammation. J Immunol. (2013) 191:1021–8. 10.4049/jimmunol.1300738 23817419

[21] Sauer KA Scholtes P Karwot R Finotto S. Isolation of CD4+ T cells from murine lungs: a method to analyze ongoing immune responses in the lung. Nat Protoc. (2006) 1:2870–5. 10.1038/nprot.2006.435 17406546

[22] Kim TS DeKruyff RH Rupper R Maecker HT Levy S Umetsu DT. An ovalbumin-IL-12 fusion protein is more effective than ovalbumin plus free recombinant IL-12 in inducing a T helper cell type 1-dominated immune response and inhibiting antigen-specific IgE production. J Immunol. (1997) 158:4137–44. 9126973

[23] Inaba K Inaba M Romani N Aya H Deguchi M Ikehara S. Generation of large numbers of dendritic cells from mouse bone marrow cultures supplemented with granulocyte/macrophage colony-stimulating factor. J Exp Med. (1992) 176:1693–702. 10.1084/jem.176.6.1693 1460426 PMC2119469

[24] Lee A Kim MS Cho D Jang KK Choi SH Kim TS. *Vibrio vulnificus* RtxA is a major factor driving inflammatory T helper type 17 cell responses *in vitro* and *in vivo*. Front Immunol. (2018) 9:2095. 10.3389/fimmu.2018.02095 30283443 PMC6157323

[25] Robinson DS. The role of the T cell in asthma. J Allergy Clin Immunol. (2010) 126:1081–91. 10.1016/j.jaci.2010.06.025 20709383

[26] Palomares O Yaman G Azkur AK Akkoc T Akdis M Akdis CA. Role of Treg in immune regulation of allergic diseases. Eur J Immunol. (2010) 40:1232–40. 10.1002/eji.200940045 20148422

[27] Martin H Taube C Regulatory T cells and regulation of allergic airway diseases. Am J Clin Exp Immunol. (2012) 1:166–78. 23885322 PMC3714190

[28] Kearley J Barker JE Robinson DS Lloyd CM. Resolution of airway inflammation and hyperreactivity after *in vivo* transfer of CD4+CD25+ regulatory T cells is interleukin 10 dependent. J Exp Med. (2005) 202:1539–47. 10.1084/jem.20051166 16314435 PMC1350743

[29] Mohamadzadeh M Olson S Kalina WV Ruthel G Demmin GL Warfield KL. Lactobacilli activate human dendritic cells that skew T cells toward T helper 1 polarization. Proc Natl Acad Sci USA. (2005) 102:2880–5. 10.1073/pnas.0500098102 15710900 PMC549474

[30] Chuang L Wu KG Pai C Hsieh PS Tsai JJ Yen JH. Heat-killed cells of lactobacilli skew the immune response toward T helper 1 polarization in mouse splenocytes and dendritic cell-treated T cells. J Agric Food Chem. (2007) 55:11080–6. 10.1021/jf071786o 18038979

[31] Kwon HK Lee CG So JS Chae CS Hwang JS Sahoo A. Generation of regulatory dendritic cells and CD4+Foxp3+ T cells by probiotics administration suppresses immune disorders. Proc Natl Acad Sci USA. (2010) 107:2159–64. 10.1073/pnas.0904055107 20080669 PMC2836639

[32] Francisco LM Salinas VH Brown KE Vanguri VK Freeman GJ Kuchroo VK. PD-L1 regulates the development, maintenance, and function of induced regulatory T cells. J Exp Med. (2009) 206:3015–29. 10.1084/jem.20090847 20008522 PMC2806460

[33] Ruane DT Lavelle EC. The role of CD103? dendritic cells in the intestinal mucosal immune system. Front Immunol. (2011) 2:25. 10.3389/fimmu.2011.00025 22566815 PMC3342356

[34] Shiokawa A Kotaki R Takano T Nakajima-Adachi H Hachimura S. Mesenteric lymph node CD11b– CD103+ PD-L1High dendritic cells highly induce regulatory T cells. Immunology. (2017) 152:52–64. 10.1111/imm.12747 28423181 PMC5543494

[35] Scott CL Aumeunier AM Mowat AM. Intestinal CD103+ dendritic cells: master regulators of tolerance? Trends Immunol. (2011) 32:412–9. 10.1016/j.it.2011.06.003 21816673

[36] Coombes JL Siddiqui KR Arancibia-Cárcamo CV Hall J Sun CM Belkaid Y. A functionally specialized population of mucosal CD103+ DCs induces Foxp3+ regulatory T cells via a TGF-β and retinoic acid-dependent mechanism. J Exp Med. (2007) 204:1757–64. 10.1084/jem.20070590 17620361 PMC2118683

[37] Fukaya T Takagi H Sato Y Sato K Eizumi K Taya H Crucial roles of B7-H1 and B7-DC expressed on mesenteric lymph node dendritic cells in the generation of antigen-specific CD4+Foxp3+ regulatory T cells in the establishment of oral tolerance. Blood. (2010) 16:2266–76. 10.1182/blood-2009-10-250472 PMC3368550 20574047

[38] Maldonado RA von Andrian UH. How tolerogenic dendritic cells induce regulatory T cells. Adv Immunol. (2010) 108:111–65. 10.1016/B978-0-12-380995-7.00004-5 21056730 PMC3050492

[39] Forsythe P Inman MD Bienenstock J. Oral treatment with live *Lactobacillus reutri* inhibits the allergic airway response in mice. Am J Respir Crit Care Med. (2007) 175:561–9. 10.1164/rccm.200606-821OC 17204726

[40] Jang SO Kim HJ Kim YJ Kang MJ Kwon JW Seo JH. Asthma prevention by Lactobacillus rhamnosus in a mouse model is associated with CD4+CD25+Foxp3+ T cells. Allergy Asthma Immunol Res. (2012) 4:150–6. 10.4168/aair.2012.4.3.150 22548208 PMC3328732

[41] Zhang J Su H Li Q Wu H Liu M Huang J. Oral administration of *Clostridium butyricum* CGMCC0313-1 inhibits β-lactoglobulin-induced intestinal anaphylaxis in a mouse model of food allergy. Gut Pathog. (2017) 9:11. 10.1186/s13099-017-0160-6 28250847 PMC5322677

[42] Segawa S Hayashi A Nakakita Y Kaneda H Watari J Yasui H. Oral Administration of heat-killed *Lactobacillus brevis* SBC8803 ameliorates the development of dermatitis and inhibits immunoglobulin E production in atopic dermatitis model NC/Nga Mice. Biol Pharm Bull. (2008) 31:884–9. 10.1248/bpb.31.884 18451512

[43] Steinman RM Hawiger D Nussenzweig MC. Tolerogenic dendritic cells. Annu Rev Immunol. (2003) 21:685–711. 10.1146/annurev.immunol.21.120601.141040 12615891

[44] Mayer CT Berod L Sparwasser T. Layers of dendritic cell-mediated T cell tolerance, their regulation and the prevention of autoimmunity. Front Immunol. (2012) 3:183. 10.3389/fimmu.2012.00183 22783257 PMC3388714

[45] Shurin MR Naiditch H Zhong H Shurin GV. Regulatory dendritic cells: new targets for cancer immunotherapy. Cancer Biol Ther. (2011) 11:988–92. 10.4161/cbt.11.11.15543 21474998

[46] Chae CS Kwon HK Hwang JS Kim JE Im SH. Prophylactic effect of probiotics on the development of experimental autoimmune myasthenia gravis. PLoS ONE. (2012) 7:e52119. 10.1371/journal.pone.0052119 23284891 PMC3527378

[47] Sun CM Hall JA Blank RB Bouladoux N Oukka M Mora JR Small intestine lamina propria dendritic cells promote *de novo* generation of Foxp3 Treg cells via retinoic acid. J Exp Med. (2007) 204:1775–85. 10.1084/jem.20070602 17620362 PMC2118682

[48] Matteoli G Mazzini E Iliev ID Mileti E Fallarino F Puccetti P. Gut CD103+ dendritic cells express indoleamine 2,3-dioxygenase which influences T regulatory/T effector cell balance and oral tolerance induction. Gut. (2010) 59:595–604. 10.1136/gut.2009.185108 20427394

[49] Hansen G Berry G DeKruyff RH Umetsu DT. Allergen-specific Th1 cells fail to counterbalance Th2 cell-induced airway hyperreactivity but cause severe airway inflammation. J Clin Invest. (1999) 103:175–83. 10.1172/JCI5155 9916129 PMC407883

[50] Stock P Akbari O Berry G Freeman GJ Dekruyff RH Umetsu DT. Induction of T helper type 1-like regulatory cells that express Foxp3 and protect against airway hyper-reactivity. Nat Immunol. (2004) 5:1149–56. 10.1038/ni1122 15448689

[51] Akbari O DeKruyff RH Umetsu DT. Pulmonary dendritic cells producing IL-10 mediate tolerance induced by respiratory exposure to antigen. Nat Immunol. (2001) 2:725–31. 10.1038/90667 11477409

[52] Zuany-Amorim C Sawicka E Manlius C Le Moine A Brunet LR Kemeny DM. Suppression of airway eosinophilia by killed *Mycobacterium vaccae*-induced allergen-specific regulatory T-cells. Nat Med. (2002) 8:625–9. 10.1038/nm0602-625 12042815

[53] Verma R Lee C Jeun EJ Yi J Kim KS Ghosh A. Cell surface polysaccharides of Bifidobacterium bifidum induce the generation of Foxp3+regulatory T cells. Sci Immunol. (2018) 3:eaat6975. 10.1126/sciimmunol.aat6975 30341145

[54] Round JL Mazmanian SK. Inducible Foxp3+ regulatory T-cell development by a commensal bacterium of the intestinal microbiota. Proc Natl Acad Sci USA. (2010) 107:12204–9. 10.1073/pnas.0909122107 20566854 PMC2901479

[55] Lee TY Kim DJ Won JN Lee IH Sung MH Poo H. Oral administration of poly-γ-glutamate ameliorates atopic dermatitis in Nc/Nga mice by suppressing Th2-biased immune response and production of IL-17A. J Invest Dermatol. (2014) 134:704–11. 10.1038/jid.2013.389 24025551

