# Supplementary material for: Staphylococcus succinus 14BME20 Prevents Allergic Airway Inflammation by Induction of Regulatory T Cells via Interleukin-10
Source: Front Immunol. 2019 Jun 4;10:1269. doi: 10.3389/fimmu.2019.01269 (PMC6559308; doi:10.3389/fimmu.2019.01269)
Supplement: Supplementary file 1 [file Data_Sheet_1.docx]

*Supplementary Material*

# Supplementary Data


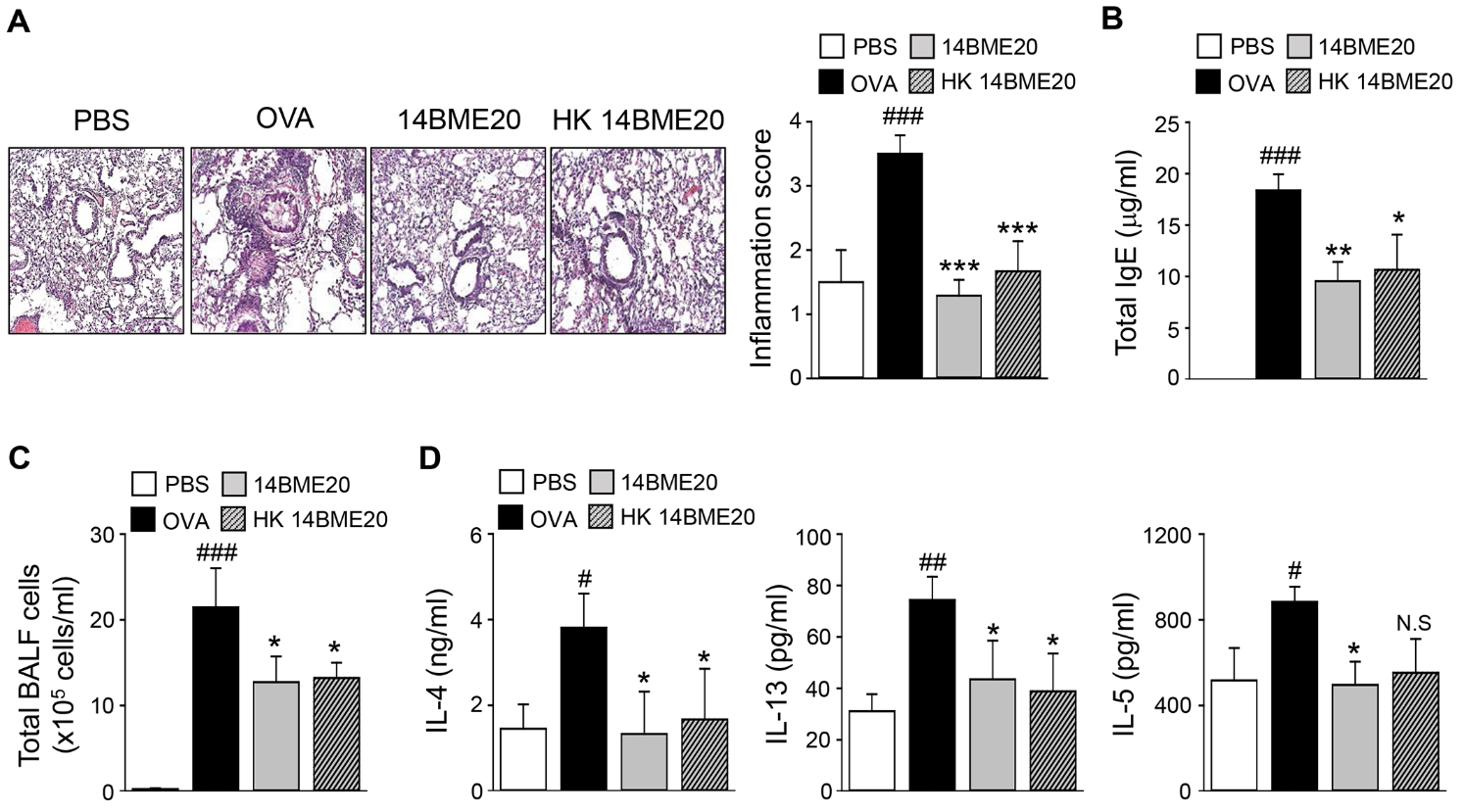


**Supplementary Figure 1. Administration of heat-killed 14BME20 inhibits allergic airway inflammation in mice.** Mice were orally administered with live 14BME20 or heat-killed 14BME20 or PBS, and then sensitized and challenged with OVA. **(A)** The lung sections were stained with hematoxylin and eosin (original magnification 100x). The scale bar shows 500 μm. The inflammatory score was assessed by measuring the infiltration of inflammatory cells into the peribronchial and perivascular tissues. **(B)** The level of IgE in sera was measured by ELISA. **(C)** Total number of inflammatory cells in BAL fluid was counted by using trypan blue stain. **(D)** The levels of Th2 cytokines in the BAL fluid were measured by ELISA. Data are representative of two independent experiments, and bar graphs represent means ± SEM. ###p<0.001, ##p<0.01, #p<0.05 versus the PBS group, ***p<0.001, **p<0.01, *p<0.05 versus the OVA group. N.S; not significant.

**
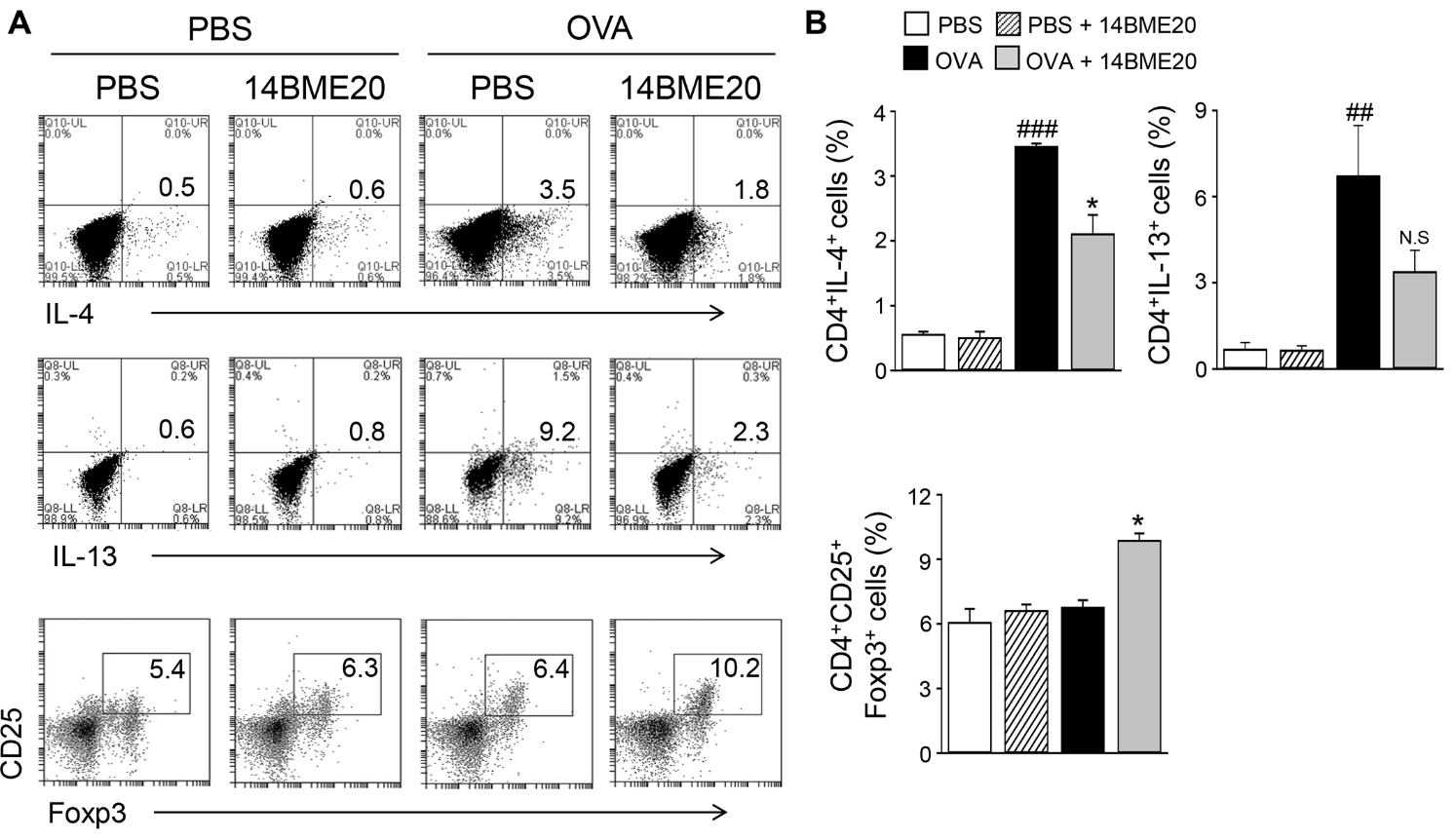
**

**Supplementary Figure 2. 14BME20 administration suppresses Th2 response and increases Treg response only when the mice are exposed to an allergen.** Mice were orally administered with 14BME20 or PBS, and then sensitized and challenged with OVA or PBS. CD4^+^ T cells were isolated from the lungs and re-stimulated for 4 days with OVA (100 μg/ml). CD4^+^ T cell subsets were analyzed by flow cytometry. Data are representative of two independent experiments, and bar graphs represent means ± SEM. ###p<0.001, ##p<0.01 versus the PBS group, *p<0.05 versus the OVA group. N.S; not significant.


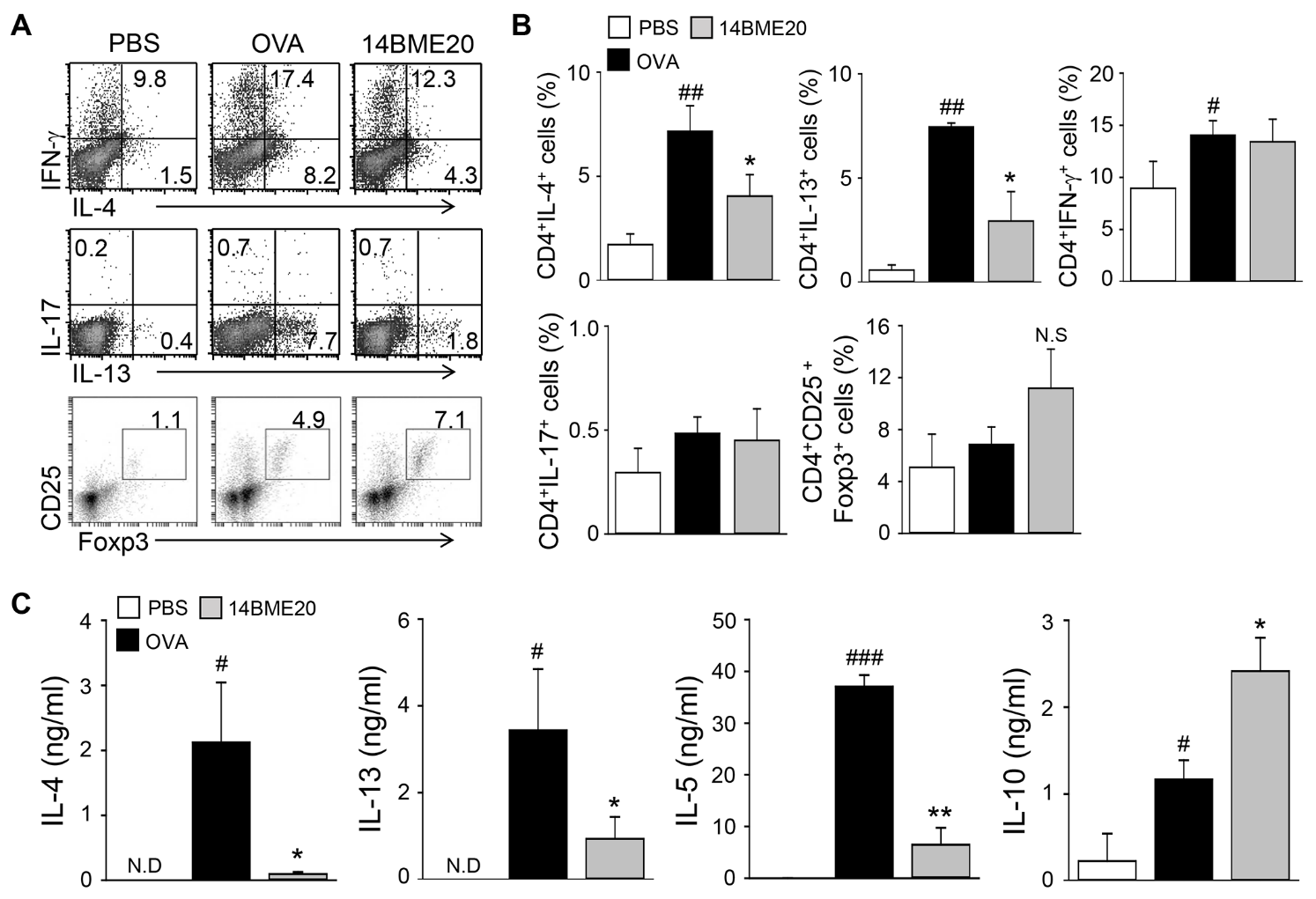


**Supplementary Figure 3. 14BME20 administration affects Th cell-mediated immune responses in spleens during allergic airway inflammation.** Mice fed with PBS or 14BME20 were challenged with OVA, and CD4^+^ T cells were isolated from spleens and re-stimulated for 4 days with OVA (100 μg/ml). **(A, B)** CD4^+^ T cell subsets were analyzed by flow cytometry. **(C)** The levels of cytokines in the supernatants were measured by ELISA. Data are representative of three independent experiments, and bar graphs represent means ± SEM. ###p<0.001, ##p<0.01, #p<0.05 versus the PBS group. **p<0.01, *p<0.05 versus the OVA group. N.D; not detect, N.S; not significant.


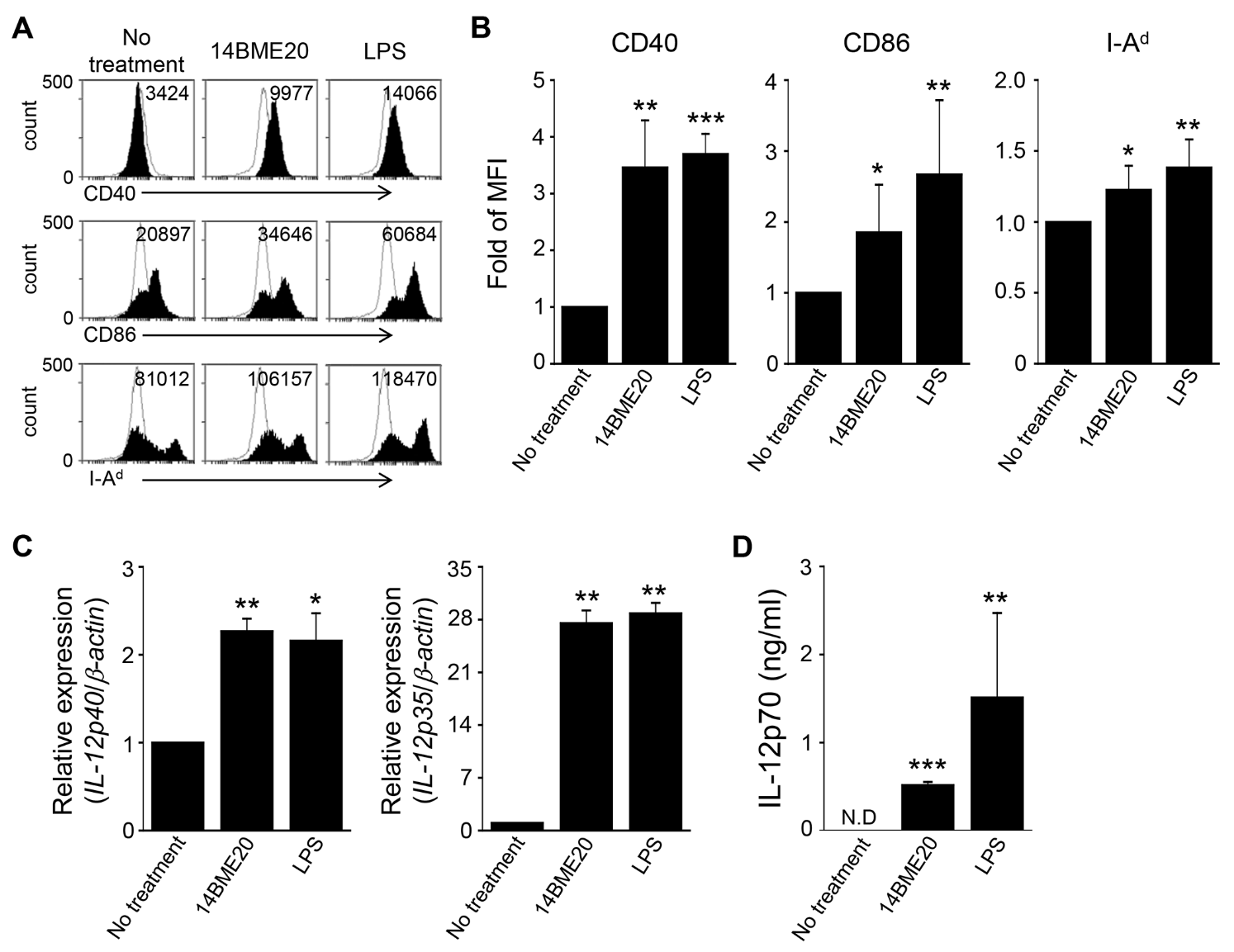


**Supplementary Figure 4. 14BME20 affects the activation of DCs.** Immature DCs were treated with 14BME20 (MOI 10) or LPS (100 ng/ml) for 24 h. **(A, B)** Expression of the activation markers on DCs was determined by flow cytometry. **(C)** The expression of IL-12p40 and IL-12p35 was analyzed by qRT-PCR. **(D)** The level of IL-12p70 was measured by ELISA. Data are representative of three independent experiments, and bar graphs represent means ± SEM. ***p<0.001, **p<0.01, *p<0.05 versus the negative control group (no treatment). N.D; not detect.


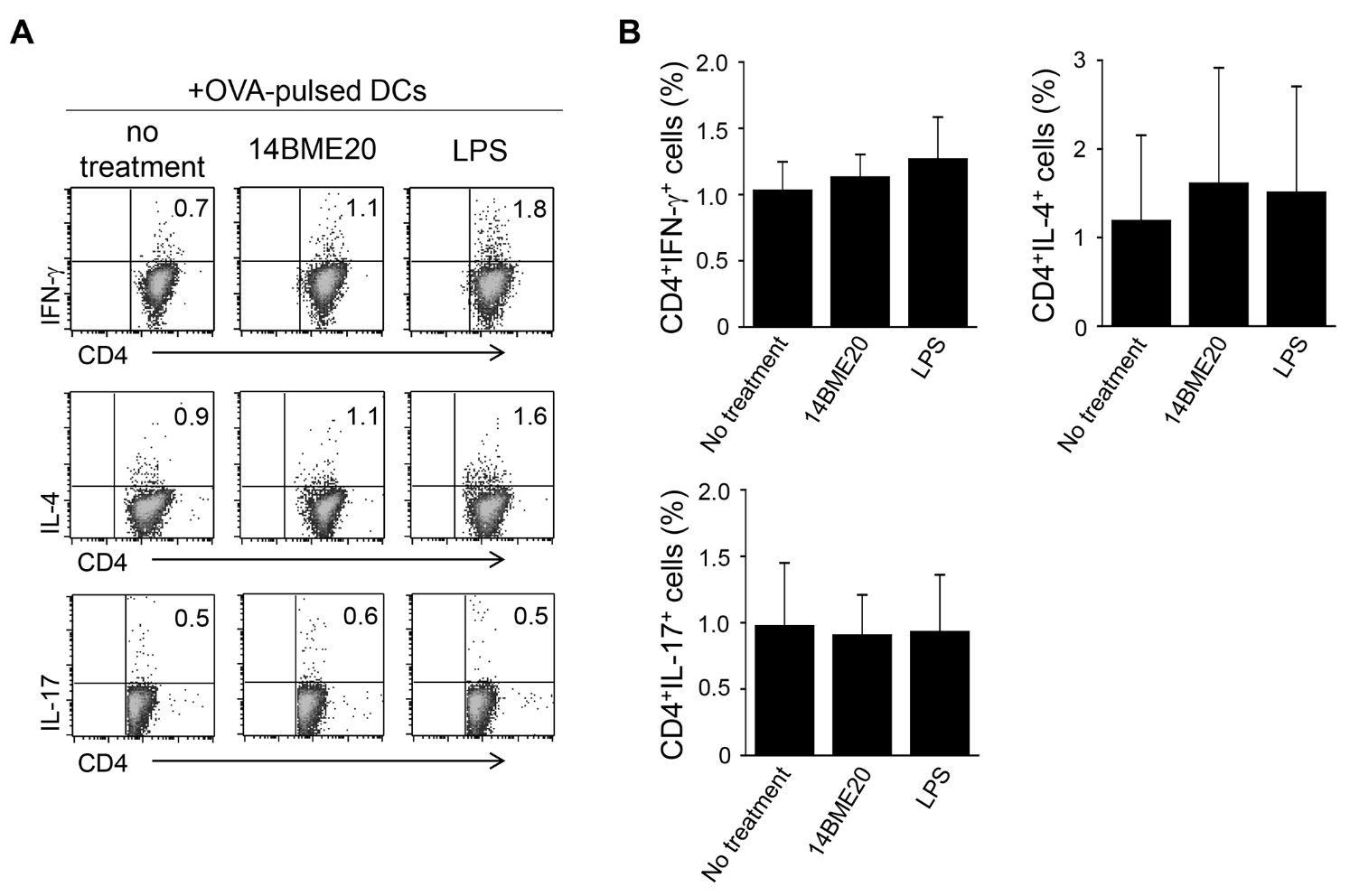


**Supplementary Figure 5. 14BME20 does not affect the population of Th1, Th2, and Th17 cells.** OVA-pulsed DCs were treated with 14BME20 or LPS for 24 h, followed by coculture with naïve CD4^+^ T cells for 4 days. The populations of CD4^+^ IFN-γ^+^ cells, CD4^+^IL-4^+^ cells, and CD4^+^IL-17^+^ cells were analyzed by flow cytometry. Data are representative of three independent experiments, and bar graphs represent means ± SEM.


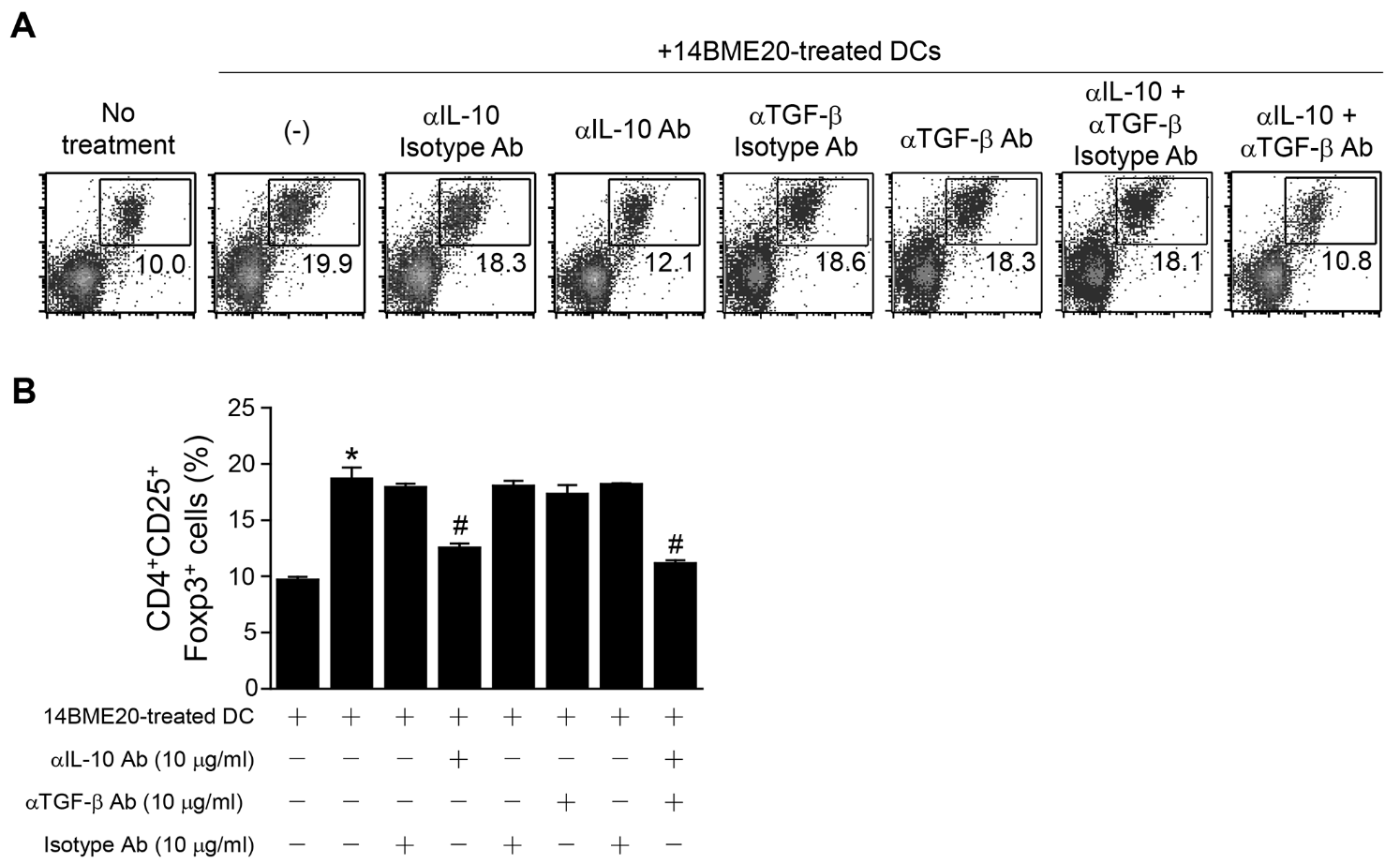


**Supplementary Figure 6. Treg cell population increased by 14BME20 is not due to TGF-β.** The OVA-pulsed DCs treated with 14BME20 were incubated for 30 min with neutralizing anti-IL-10 mAb and/or anti-TGF-β mAb, followed by coculture for 4 days with naive CD4^+^ T cells. The percentage of Treg cell population was assessed by flow cytometry. Data are representative of three independent experiments, and bar graphs represent means ± SEM. *p<0.05 versus the negative control (no treatment). #p<0.05 versus the 14BME20-treated group.
